# A Comparative Study of Control Approaches in Hybrid Reinforcement Learning-Based Drone Swarms

**DOI:** 10.3390/s26144395

**Published:** 2026-07-10

**Authors:** Raúl Arranz, Juan A. Besada, David Carramiñana

**Affiliations:** Information Processing and Telecommunications Center, Universidad Politécnica de Madrid, ETSI Telecomunicación, Av. Complutense 30, 28040 Madrid, Spain; raul.arranz@upm.es (R.A.); d.carraminana@upm.es (D.C.)

**Keywords:** hybrid-AI, deep reinforcement learning, drone swarm control

## Abstract

Reinforcement learning (RL) has emerged as a powerful paradigm for enabling autonomous coordination in multi-UAV systems operating in complex and uncertain environments. However, the effectiveness of learned policies is strongly influenced by how actions are implemented at the control level, an aspect that has received limited attention in the literature. This paper presents a comparative study of three control methods (heading-based, waypoint-based, and deterministic) within a unified hybrid-AI architecture, in which the same RL policy structure is used across two of the three configurations. By isolating the control method as the sole variable, the study evaluates how different action abstractions affect learning efficiency, robustness, and operational performance in cooperative surveillance missions. A statistically rigorous Monte Carlo evaluation, supported by non-parametric hypothesis testing, demonstrates that heading-based control consistently achieves superior performance in terms of revisit period, target acquisition time, and tracking continuity. The analysis further reveals that these gains arise from improved reactivity and constraint handling rather than from differences in policy learning. The results highlight the critical role of control-level design in RL-based multi-agent systems and provide practical guidelines for selecting action abstractions in aerial swarm applications.

## 1. Introduction

Unmanned aerial vehicle (UAV) swarms are increasingly envisioned as a key technological enabler for applications such as persistent surveillance [1], search and rescue missions [2], payload transportation [3], reconnaissance and mapping [4], and public communication initiatives [5]. These scenarios are characterized by high levels of uncertainty, partial observability, dynamic environments, and complex interactions among multiple agents, making traditional rule-based or centralized trajectory-planning approaches difficult to scale and maintain.

In this context, reinforcement learning (RL) has shown remarkable potential as a data-driven approach capable of learning adaptive behaviors directly through interaction with the environment. RL-based methods are particularly attractive for multi-UAV control systems due to their ability to cope with partially observable state spaces, dynamically changing conditions, and decentralized decision-making. Recent advances have demonstrated promising results in multi-agent coordination, collision avoidance, and cooperative task execution.

Despite this progress, the performance of RL in real-world multi-agent systems does not depend solely on the learning algorithm itself. Instead, it is critically shaped by several design choices that define how the agent interacts with the environment, most notably the structure of the action space, the observation representation, and the decision frequency at which policies are executed. These elements determine the temporal and spatial resolution of control, directly affecting reactivity, stability, and constraint satisfaction.

While RL has shown remarkable potential in multi-UAV coordination, the impact of the underlying control abstraction on learning effectiveness remains underexplored. In many existing works, the choice of control representation (whether actions correspond to low-level velocities, intermediate waypoints, or heading commands) is treated as an implementation detail rather than as a fundamental design decision. As a result, it remains unclear to what extent observed performance differences are attributable to the learning process itself or to the control abstraction through which learned actions are implemented.

This paper addresses this gap by conducting a systematic comparison of two RL-based hybrid-AI control methods and a purely deterministic control method within a unified experimental framework. In the first two methods, a single RL policy architecture and training procedure are employed, while the downstream control method responsible for translating policy outputs into motion commands is varied. This approach enables a fair and isolated evaluation of how different action abstractions influence learning dynamics and operational performance. The comparison is carried out in cooperative surveillance scenarios using a large-scale Monte Carlo simulation framework complemented by statistical hypothesis testing to ensure robust conclusions.

By explicitly focusing on the interaction between RL and control method design, this work contributes to a deeper understanding of how hybrid-AI systems should be structured for reliable and efficient multi-UAV operation.

## 2. Related Works

Reinforcement learning has emerged as a powerful paradigm for enabling autonomous coordination in multi-UAV systems operating in dynamic, partially observable, and large-scale environments. Unlike classical optimization and model-based control techniques, which require explicit modeling and often struggle with high-dimensional interactions, RL allows agents to learn policies directly from environmental feedback. This property makes it particularly attractive for swarm missions involving cooperative search, persistent tracking, and distributed sensing.

### 2.1. Reinforcement Learning for Multi-UAV Systems

In multi-agent settings, however, RL introduces additional complexity due to non-stationarity: as originally noted in early multi-agent studies [6,7], each agent’s learning process alters the environment perceived by the others. To address this issue, Multi-Agent Reinforcement Learning (MARL) has evolved along several complementary directions. Centralized Training with Decentralized Execution (CTDE) has become a dominant framework, leveraging global information during training while maintaining decentralized policies during execution [8,9]. Value-decomposition approaches such as VDN [10] and QMIX [11] enable scalable coordination through the structured factorization of global value functions, whereas actor–critic and policy-gradient methods extend continuous-control algorithms such as PPO to multi-agent domains [12].

Beyond coordination mechanisms, research has incorporated communication learning [13,14], relational inductive biases through graph-based models [15], and mean-field approximations for large homogeneous populations [16,17]. In UAV swarm contexts, MARL has been applied to energy-aware trajectory optimization [18], safe navigation with collision filtering [19], robust communication under channel disruptions [20], and joint sensing–communication optimization [21]. Collectively, these works demonstrate the versatility of MARL for aerial multi-agent systems.

However, most existing contributions focus primarily on algorithmic stability, reward engineering, communication strategies, or safety filtering. The structure of the control interface through which the learned policy affects vehicle motion is typically treated as a fixed implementation detail rather than as a factor influencing learning dynamics.

### 2.2. Curriculum Learning in Multi-Agent RL

Training stability and generalization remain central challenges in MARL, particularly in continuous-control UAV environments characterized by large state–action spaces and strong inter-agent coupling. Curriculum learning (CL) has therefore emerged as a structured strategy to mitigate non-stationarity and improve convergence.

The core idea of CL is to organize training into progressively more complex stages, gradually increasing task difficulty as agents acquire competence. This staged paradigm has been shown to improve stability and sample efficiency in multi-agent environments, including the widely adopted StarCraft Multi-Agent Challenge (SMAC) benchmark [22]. In UAV swarm applications, curriculum-based approaches have demonstrated improved formation maintenance and obstacle avoidance by introducing environmental complexity incrementally [23].

Recent research has explored automatic curriculum generation mechanisms that adapt task difficulty dynamically according to agent performance [24]. Moreover, curriculum learning has been combined with heterogeneous population training strategies such as Skilled Population Curriculum (SPC) [25] and has been identified as a key enabler for transfer and lifelong learning in multi-agent systems [26]. By exposing agents to a spectrum of progressively challenging scenarios, CL enhances policy robustness and reduces overfitting to narrow environmental conditions.

Despite these advances, most curriculum-based MARL studies assume a fixed action and observation parameterization throughout training. The interaction between curriculum progression and control abstraction, particularly in continuous UAV guidance problems, remains largely unexplored.

### 2.3. Control Representation in Learning-Based UAV Systems

In multi-UAV systems, the learning policy can interface with the platform at different levels of control abstraction, which fundamentally shapes the structure of the action space and the temporal resolution of decision-making. This interface can be implemented through different control paradigms, including:

- High-level control (waypoint-based), where policies output spatial targets in global coordinates that are subsequently tracked by lower-level controllers [27,28].
- Low-level control (attitude/thrust-based), where actions directly parameterize motion primitives such as commanded attitude angles and thrust inputs [29,30].
- Deterministic guidance laws, where trajectories are generated by predefined algorithms without learning [31].

Although these representations differ fundamentally in their temporal granularity, expressiveness, and sensitivity to disturbances, the majority of RL-based UAV studies adopt a single control interface without systematically evaluating its impact on learning dynamics. The choice of control abstraction directly affects:

- The dimensionality and geometry of the action space.
- The smoothness of the policy–environment mapping.
- The frequency at which corrective decisions can be issued.
- The ease with which safety constraints can be incorporated.

In continuous-control RL, such factors can significantly influence gradient estimation, exploration efficiency, reward propagation, and, ultimately, convergence stability. Nevertheless, comparative analyses isolating the effect of control representation within a unified RL framework remain scarce.

Recent learning-based UAV control research has also explored advanced guidance and formation-control strategies beyond conventional waypoint- or velocity-command representations. Examples include Finite-Time Learning-Based Optimal Elliptical Encirclement Control for UAVs With Prescribed Constraints [32], which incorporates prescribed-performance constraints, adaptive estimation mechanisms, and stability guarantees. Such approaches have demonstrated strong performance in cooperative UAV missions, particularly for fixed-wing formations and target encirclement tasks. However, these methods address different operational objectives and vehicle dynamics from those considered in this work.

The present study focuses on multirotor UAV swarms performing cooperative surveillance missions that require continuous transitions between area search and target-tracking task execution. Rather than proposing a new RL algorithm, our objective is to isolate the effect of the control abstraction itself, independently of the learning architecture, and to quantify how different action representations influence the resulting swarm behavior.

### 2.4. Positioning of This Work

Although RL has shown remarkable potential in multi-UAV coordination, the impact of the underlying trajectory-control abstraction on learning effectiveness remains underexplored. In most studies, the action representation is selected as an implementation detail rather than treated as a variable influencing policy optimization. However, from a learning-theoretic perspective, the control parameterization shapes the topology of the action space, the temporal resolution of decisions, and the smoothness of the reward landscape. These factors directly affect exploration efficiency, gradient variance, policy expressiveness, and convergence stability. Consequently, different control abstractions may yield substantially different learning behaviors even when trained under identical reward functions and environmental conditions. A systematic comparison isolating this effect is largely absent from the UAV swarm literature.

This paper addresses this gap by systematically comparing two reinforcement learning-based hybrid-AI control methods and a purely deterministic control method within a common experimental framework. By maintaining identical reward structures, observation models, training procedures, and evaluation methodology, the study isolates the impact of control abstraction on learning effectiveness and mission performance.

Rather than proposing a new MARL algorithm, the contribution lies in demonstrating how the structure of the control interface itself influences convergence speed, robustness, search efficiency, and tracking quality in multi-UAV swarm operations.

## 3. RL-Based Hybrid Architecture

The proposed system follows a hierarchical hybrid-AI architecture that decouples high-level mission coordination from low-level motion generation. This separation enables scalable swarm management while allowing reinforcement learning (RL) to govern agent-level behavior within clearly defined operational constraints.

**Figure 1:**
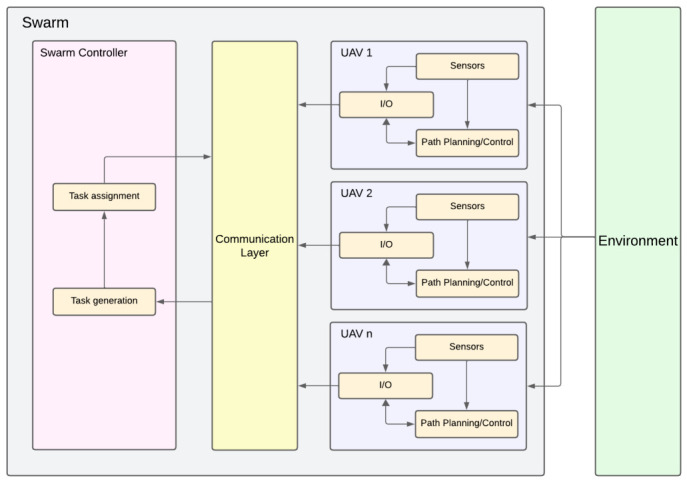
General architecture of the swarm control process.

The architecture is structured into two decision layers: a centralized swarm controller (SC) responsible for task allocation and decentralized RL-based agents responsible for trajectory execution (Figure 1).

### 3.1. System Overview

Ground surveillance constitutes a demanding benchmark for cooperative multi-UAV systems, as it combines systematic area coverage with persistent monitoring of dynamic targets under operational constraints. Unlike narrowly scoped missions such as inspection or delivery, surveillance requires continuous adaptation to partially observable environments, dynamic target behavior, and potential sensing or communication disruptions. These characteristics make it a suitable testbed for evaluating RL-based control methods within a hybrid architecture.

The mission is defined over a bounded three-dimensional operational volume, characterized by polygonal ground limits and altitude constraints. Within this area, a swarm of multirotor UAVs is tasked with two primary objectives: (i) systematic exploration of the operational region to ensure timely coverage and revisits, and (ii) continuous tracking of ground targets once detected. These objectives introduce a dual requirement: efficient spatial exploration and responsive, real-time adaptation to target motion.

To enable scalability and modularity, the surveillance mission is decomposed into two fundamental tasks: search and tracking. The search task focuses on systematic area reconnaissance, ensuring adequate spatial coverage while minimizing redundancy and energy consumption. The tracking task is activated upon target detection and aims to maintain persistent observation, including target reacquisition in the case of temporary occlusions or losses. At the swarm level, a coordination mechanism dynamically allocates drones between these tasks, ensuring that coverage and persistence are maintained without overcommitting resources.

The system follows a hybrid architecture in which reinforcement learning governs agent-level decision-making, while deterministic modules guarantee execution stability and safety. RL policies are responsible for generating motion commands according to the selected control abstraction, whereas lower-level controllers handle trajectory execution. This separation allows learning-based adaptability while preserving operational reliability. As the platforms considered throughout this study are multirotor UAVs, this lower-level loop benefits from the well-established stability properties of multirotor flight (decoupled, near-omnidirectional translational dynamics, no stall or minimum-airspeed constraints), typically guaranteed via standard cascaded PID or geometric attitude/velocity controllers. This inner loop is identical across the compared control methods. A formal Lyapunov-style stability or constraint-satisfaction analysis of this loop is consequently outside the scope of the present comparison. Such analysis becomes substantially more critical for platforms with coupled, underactuated dynamics, such as fixed-wing UAVs subject to stall and minimum-airspeed limits. Therefore, extending our results to those platforms is not straightforward, and analyzing them may be a relevant direction for future work.

All training and evaluation procedures are conducted in simulation. A three-dimensional physics-based environment developed in Unity is used to model the operational area, drone dynamics, sensing mechanisms, and target behavior. The ML-Agents toolkit [33] provides the interface between the simulation and the RL training pipeline, enabling standardized policy learning using Proximal Policy Optimization (PPO) [34]. Each drone is modeled as an autonomous agent equipped with perception modules that provide target and obstacle information, subject to range limitations and occlusion effects.

The simulation framework ensures full control over environmental conditions, repeatability of experiments, and scalable multi-agent training, thereby enabling a statistically rigorous comparison of the evaluated control methods.

### 3.2. Swarm-Level Coordination

**Figure 2:**
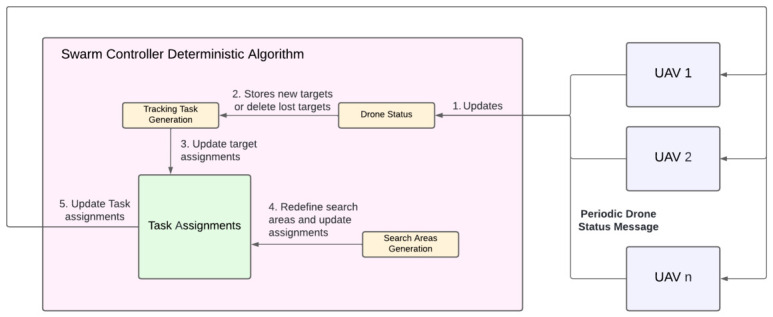
Rule-based algorithm followed by the SC.

At the upper layer (described in Figure 2), the swarm controller maintains a fused common operational picture (COP) constructed from periodic status messages received from all drones. The COP aggregates the state of each drone, detected targets (with fused position and velocity estimates), current task assignments, and the partition of the operational area into search regions.

Based on this operational picture, the SC executes a rule-based task-allocation procedure. Tasks are defined at a semantic level and consist of either systematic area exploration (search) or persistent monitoring of a detected target (tracking). The controller determines which drone performs which task, but it does not generate trajectories or compute motion primitives.

Task allocation follows a set of explicit operational constraints. Each drone can execute only one task at a time, and each search region or tracked target is assigned to at most one drone. Tracking tasks take precedence over search tasks, ensuring that newly detected targets are immediately serviced. At the same time, full coverage of the operational area must be preserved: the search regions dynamically adapt so that their union continuously spans the mission space, and at least one drone remains allocated to exploration. Whenever new targets are detected, the controller re-evaluates assignments and redistributes search areas among the remaining available drones.

This design ensures mission-level coherence while preventing redundant allocations and guaranteeing that both coverage and tracking objectives are maintained.

### 3.3. RL-Based Agent Layer

The lower layer consists of decentralized agents, one per drone, responsible for translating assigned tasks into motion commands.

Rather than training a single monolithic policy for the full surveillance mission, the problem is decomposed into two task-specialized RL policies: one for search and one for tracking. Upon receiving a task from the controller, the agent activates the corresponding policy.

Each policy operates primarily based on local observations, including the drone state and onboard sensor detections. However, agents also receive global information regarding target detections through the common operational picture maintained by the swarm controller. This shared situational awareness enables coordinated responses to detected targets while avoiding full state sharing among agents. The overall architecture thus preserves a modular decomposition: high-level coordination and information fusion are handled deterministically by the supervisory controller, whereas motion generation is learned through RL policies. This design reduces the effective complexity of the learning problem while still enabling cooperative behavior across the swarm.

The RL-based control methods evaluated share the same swarm controller, RL network architecture, observation structure, reward formulation, and training procedure. For each method, two distinct policies are trained, corresponding to the search and tracking tasks. The only difference between the methods lies in the trajectory-planning layer that interprets and executes the RL actions. The deterministic approach also shares the same swarm controller and observation structure but replaces the learned policies with predefined control laws.

This architectural invariance is critical: it ensures that any performance differences observed in the experimental results can be attributed exclusively to the adopted control abstraction, rather than to variations in coordination logic, reward shaping, or policy design.

## 4. Control Methods as Action Abstraction

The three control approaches implemented and evaluated in this study are described in this section.

### 4.1. High-Level Control: Waypoint-Based RL

In the waypoint-based strategy, the RL model output is interpreted as the coordinates of the next waypoint in the global reference frame. This predicted waypoint serves as an intermediate navigation target that the drone must reach by following a straight-line trajectory at constant maximum speed. The drone executes low-level movement commands that direct it toward the waypoint, and once the waypoint is reached, a new one is calculated based on updated observations from the environment. Therefore, the decision-making period is not constant; it depends on the distance between consecutive waypoints.

This method allows the policy to encode long-term planning behavior by predicting positions that may be spatially distant from the drone’s current location. Consequently, the model must implicitly learn to balance exploration and exploitation, anticipate the motion of dynamic ground targets, and account for the presence of static obstacles in the environment. In fact, every time a new obstacle is detected by the proximity sensor, the current movement is interrupted, the previous target waypoint is discarded and the policy model is queried for a new waypoint that takes the obstacle position into account.

In this method, the generated waypoint is not interpreted relative to the agent’s current position or orientation but is instead expressed in absolute coordinates. This simplifies the trajectory-generation process but requires the policy to learn meaningful mappings in the global coordinate space, which increases the learning complexity.

Once the waypoint is defined, the drone computes a normalized direction vector from its current position to the waypoint and moves in that direction at a fixed velocity. This decoupling of decision-making (agent or RL policy) and execution (drone motion controller) provides flexibility in controlling the drone’s behavior and facilitates integration with different navigation dynamics or safety constraints.

This strategy is particularly well-suited to tasks involving structured exploration or long-range target acquisition, where predictive spatial reasoning plays a critical role.

### 4.2. Low-Level Control: Heading/Velocity-Based Method

In contrast to the waypoint-based strategy, the heading-based trajectory-planning method interprets the output of the RL model as a motion vector that directly specifies the desired direction and speed of the drone’s movement. The direction is given by the orientation of the vector, while its magnitude determines the instantaneous speed. This control formulation allows the policy to continuously influence both the heading and velocity of the drone at each decision step.

In this approach, the drone no longer plans toward a specific waypoint in the global coordinate frame. Instead, it responds to environmental observations by adjusting its movement vector periodically, with updates occurring every 0.1 s. This enables more reactive and fine-grained adaptation of the trajectory to dynamic changes in the environment, such as the appearance of obstacles or the movement of ground targets.

One key distinction of this method is that the movement speed is not fixed, as in the waypoint-based approach, but is instead learned and dynamically adjusted by the agent. This additional control dimension allows the policy to modulate its behavior more precisely, accelerating in open areas or during search phases and slowing down in the presence of nearby obstacles or during tracking phases.

The continuous and local nature of the heading-based control makes it well-suited to real-time navigation tasks where agility and responsiveness are critical. However, it places higher demands on the policy’s ability to make stable decisions under partial observability and may require denser training feedback to learn optimal behaviors over time.

Overall, this strategy introduces greater expressiveness and adaptability into the drone’s motion generation, at the cost of increased control complexity and the need for more frequent model updates.

It should be noted that the control abstractions considered in this work inherently operate at different update rates. Heading-based control produces low-level motion commands that are updated at the flight-control frequency, enabling continuous trajectory adjustments. In contrast, waypoint-based control generates higher-level spatial objectives that remain active until a waypoint is reached or replanning is triggered. These update mechanisms are representative of common multirotor UAV implementations and are therefore considered an integral part of the corresponding control paradigms rather than an independent experimental variable.

### 4.3. Deterministic Guidance Method

As a baseline, a deterministic guidance strategy was implemented. This method generates waypoints using handcrafted geometric rules and interprets them identically to the high-level RL strategy. It does not involve learning and serves to quantify the benefits introduced by RL-based control.

During search, the planner generates a structured lawnmower coverage pattern, ensuring full sensor footprint coverage of the assigned region. The lateral spacing between sweeps is computed from the sensor field-of-view diameter to minimize redundancy while guaranteeing complete area coverage. This results in an efficient and predictable exploration trajectory.

During tracking, the planner exploits full knowledge of the target’s position and velocity to maintain the drone in a trailing configuration. Waypoints are generated to keep the target aligned with the center of the forward-facing sensor at a distance equal to three-fourths of the sensor radius. If this distance threshold is exceeded, a corrective waypoint is computed. If the target is lost, a local deterministic search around the last known position is executed.

This strategy performs reliably under ideal assumptions but lacks adaptability to uncertainty, sensing noise, or dynamic disturbances.

For both tasks, obstacle avoidance is handled by a deterministic Artificial Potential Field (APF) mechanism [35]. Obstacles generate repulsive potentials, and navigation objectives generate attractive potentials. Rather than computing continuous gradients, the UAV evaluates 30 discrete candidate directions placed on a circle of predefined radius and selects the direction associated with the lowest potential.

The scan radius regulates trajectory resolution. During search, a larger radius (50 m) prioritizes coverage efficiency. During tracking, a smaller radius (10 m) enables fine-grained adjustments.

This modular design ensures that differences in performance arise from the control abstraction rather than from variations in safety mechanisms.

It is important to note that standard APF methods can suffer from local minima and may require additional strategies to guarantee robustness in complex environments (see [36,37]). The deterministic planner is not intended to represent the state of the art in classical trajectory planning but rather to provide a transparent and widely used rule-based baseline within the same swarm architecture. The operational scenarios considered in this study contain a limited number of static obstacles and do not exhibit highly cluttered geometries in which APF-induced local minimum failures typically become dominant.

## 5. Training and Curriculum Learning

### 5.1. Training Scenario

This section describes the training setup used to learn the RL policies governing each agent. A common baseline environment is first defined, after which task-specific adaptations are introduced.

Targets of interest are ground vehicles, and therefore the operational domain is modeled as a two-dimensional space. The baseline scenario consists of:

- A 640×400 m semi-urban rectangular area containing two static buildings modeled as 15×10 m rectangular prisms with sufficient height to occlude visibility.
- UAVs flying at a fixed altitude of 45 m AGL. This simplification reduces simulation complexity and allows the problem to be treated as 2D, as the focus is on swarm control rather than aerodynamic modeling.

Each UAV follows the dynamic constraints of the Dronetools MiniCóndor platform: maximum take-off weight 7 kg, payload up to 3 kg, endurance 50 min, cruise speed 0–16 m/s (extended to 20 m/s in simulation for safety margin), climb rate 0–6 m/s, service ceiling 3000 m AMSL, and approximate spherical collision radius of 0.5 m. These constraints ensure physical consistency with real-world platforms.

Perception is modeled through a forward-facing RGB camera. A kinematic constraint enforces strictly forward motion. At 45 m altitude, the projected ground FoV has a center located 115 m ahead of the UAV and a radius of 60 m. Target visibility is determined using a Unity RayCast between UAV and target, blocking detection if an obstacle intersects the ray.

Each UAV also carries a 100 m radius proximity sensor for obstacle detection. Precise localization is assumed, and navigation errors arising from GPS uncertainty, IMU drift, or state-estimation inaccuracies are assumed to be negligible in this study, as the focus is on control and coordination rather than state-estimation uncertainty. Weather conditions are also assumed to be benign. This assumption is commonly adopted in simulation-based reinforcement learning studies that focus on UAV navigation and multi-agent coordination, where the primary objective is to evaluate decision-making and control strategies rather than localization performance [38].

Introducing localization uncertainty would add an additional source of variability that could mask the differences attributable to the control method itself. In any case, assessing robustness under realistic navigation uncertainty is considered an important direction for future work. Recent studies have specifically addressed localization degradation and GPS-denied operation in multi-UAV systems, highlighting this topic as a complementary research problem [39].

For the search model, the baseline is extended with a designated search Bbox requiring systematic area coverage.

For the tracking model, a ground vehicle is introduced. The vehicle follows straight-line trajectories between randomly sampled waypoints, generating dynamic and unpredictable conditions.

Although simplified (fixed altitude, forward-only motion, linear targets), the framework is generalizable to 3D navigation, non-linear dynamics, and more complex terrain, which remain outside this paper’s scope.

### 5.2. Action–Observation Space Structure

Two coordinate systems are used:

- Global Cartesian coordinates (x, y) for map-referenced quantities.
- Local polar coordinates (r,θ) (centered at the UAV position but aligned with the global coordinates) for direction-driven control.

Common data abstractions include global/local positions, speed vectors, bounding boxes, and proximity-based relative vectors.

Waypoint-based and deterministic control use Cartesian representations, whereas heading-based control relies on polar representations for motion commands, reflecting its direction-oriented structure.

#### 5.2.1. Observations

All models receive:

- Drone state: global position and velocity (Cartesian for waypoint-based, polar for heading-based).
- Proximity sensor data: relative positions of the two closest obstacles and closest drone (plus neighbor velocity), expressed in local coordinates. Zero padding is applied when absent.

Additional task-specific inputs are defined in Section 5.3.1 and Section 5.3.2.

Inputs are normalized (positions with respect to area size, velocities with respect to max speed). This ensures numerical stability, accelerates convergence, prevents feature dominance, and enables transferability across differently scaled environments.

Rather than training a separate obstacle-avoidance model (which would introduce action discontinuities), avoidance is integrated directly into the search and tracking policies.

#### 5.2.2. Actions

The policy outputs a 2D continuous vector shared across control paradigms:

- Waypoint-based: interpreted as a global (x,y) waypoint.
- Heading-based: interpreted as (r,θ) speed magnitude and heading.

This shared output format enables architectural consistency and fair comparison across control abstractions.

### 5.3. Curriculum Learning Training

Due to sparse rewards and large state spaces, all models are trained via a manually designed curriculum learning (CL) strategy. Training environments $E_{1},\ldots ,E_{n}$ are ordered by increasing difficulty. Progression occurs when a performance threshold (average reward over a moving window) is exceeded.

Direct training in complex environments failed to converge, whereas CL produced stable learning and reduced variance across seeds.

Curricula were designed based on prior empirical experience with multi-agent RL and validated through exploratory experiments. Early stages use strongly constrained reward definitions; later stages progressively relax constraints to promote generalization while avoiding reward hacking. After each stage, policies were evaluated in unseen scenarios to detect exploitative behaviors.

Policies are implemented as fully connected networks (input layer, two hidden layers of 128 neurons, output layer). PPO is used in all cases for its stability in continuous control. Hyperparameters are kept constant across curriculum stages.

For tasks requiring memory (e.g., search), an RNN-based architecture augments the policy to encode temporal dependencies and prevent redundant exploration.

#### 5.3.1. Search Model

The search model receives additional inputs describing the search Bbox:

- Position of Bbox center (global Cartesian for waypoint-based; local polar for heading-based).
- Bbox size (2D vector).

Five curriculum stages are defined for each control abstraction. Although both curricula progressively increase environmental complexity, their progression mechanisms differ due to the distinct action semantics of each control formulation.

- Heading-based curriculum: in this case, the cell size is kept fixed such that the projected sensor footprint fully covers a cell when its center lies inside it. Task complexity increases primarily by expanding the search area and introducing obstacles:
  $E_{1}$. 2×2 cells, max steps set to 5000.
  $E_{2}$. 3×3 cells, max steps set to 10,000.
  $E_{3}$. 4×3 cells, max steps set to 15,000.
  $E_{4}$. Area dimensions randomly varying from 3 to 5 cells, max steps set to 15,000.
  $E_{5}$. Area dimensions randomly varying from 3 to 6 cells, 4 static obstacles, max steps set to 20,000.
- Waypoint-based curriculum: due to the higher-level spatial abstraction of waypoint actions and the tendency to overshoot boundaries during early training, the search area is initially kept fixed while the grid resolution is progressively refined. Once the minimum cell size (identical to that used in the heading-based setup) is reached, complexity increases analogously by enlarging the area and adding obstacles:
  $E_{1}$. Fixed area, divided into 2×2 cells, max steps set to 5000.
  $E_{2}$. Fixed area, divided into 3×3 cells, max steps set to 10,000.
  $E_{3}$. Fixed area, divided into 4×4 cells, max steps set to 10,000 (cell size fixed from this stage onward).
  $E_{4}$. Area dimensions randomly varying from 3 to 5 cells, max steps set to 15,000.
  $E_{5}$. Area dimensions randomly varying from 3 to 6 cells, 4 static obstacles, max steps set to 20,000.

**Figure 3:**
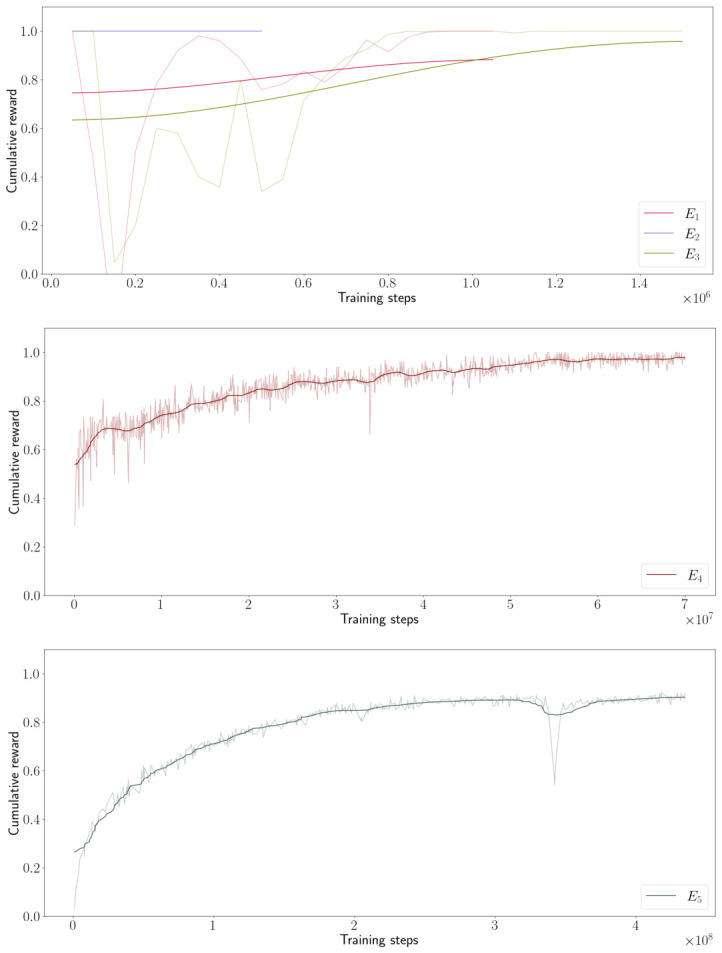
Search model cumulative reward training progress with heading-based control.

**Figure 4:**
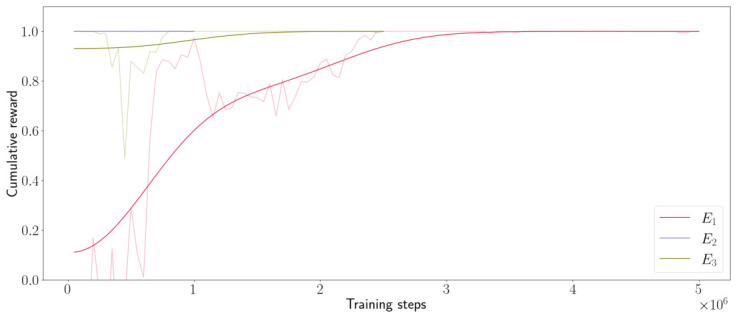

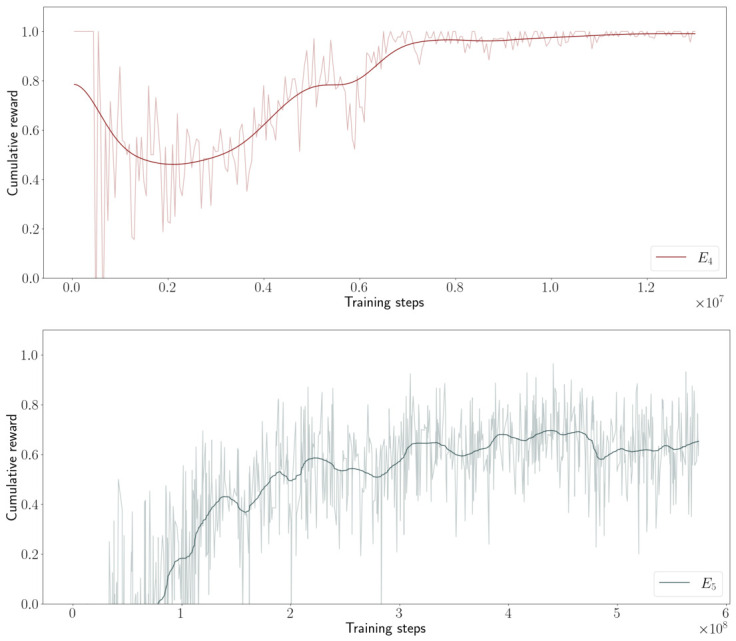
Search model cumulative reward training progress with waypoints-based control.

It is important to note that, despite their differing early-stage progression, both curricula converge to identical final scenario specifications: stages E4 and E5 are defined with the same area-size range, obstacle configuration, and step budget for both control methods, and stage E3 already matches the minimum cell size between methods. The divergence is therefore confined to the initial stages (E1–E3), whose purpose is solely to bootstrap stable convergence given the distinct failure modes empirically observed for each action semantics (e.g., systematic boundary overshooting when training the waypoint-based policy directly on a fine grid resolution). Both curricula were independently tuned until reaching comparable converged performance, as illustrated by the cumulative reward curves in Figure 3 and Figure 4. Consequently, the reported performance differences would reflect the behavior of each control method under common, previously unseen, full-complexity evaluation conditions, rather than an artifact of unequal early-stage exposure during training.

Rewards are designed so that ideal cumulative return equals 1. Penalties exceed corresponding positive rewards to prevent oscillatory exploitation. While this design may introduce a degree of risk-averse behavior, the same reward structure is applied to all RL-based control methods evaluated in this work. Consequently, the resulting trade-off between safety and mission performance affects both approaches equally and does not alter the validity of the comparative analysis. Boundary, coverage, collision, and time-limit incentives are included (Table 1).

Cumulative reward curves (Figure 3 for the heading approach and Figure 4 for the waypoint-based approach) show increasing trends with PPO-consistent fluctuations. Each color corresponds to a different curriculum step. For a given color, light lines depict the raw values while the bold ones showcase an smoothed line to better identify trends. Smoothed curves confirm stable convergence across curriculum stages.

#### 5.3.2. Tracking Model

The tracking model receives:

- The target position (global Cartesian or local polar depending on control paradigm).
- The target velocity vector.
- A Boolean flag indicating whether the target is within the FoV.

Episodes require maintaining target visibility for progressively longer consecutive steps, with increasing episode limits and environmental complexity (obstacles introduced in later stages):

$E_{1}$. 50 steps with a max step of 2000.
$E_{2}$. 250 steps with a max step of 3000.
$E_{3}$. 500 steps with a max step of 5000.
$E_{4}$. 1000 steps with a max step of 7000.
$E_{5}$. 1500 steps, placing four static obstacles alongside the operational area and setting the max step to 15,000.

The reward function is designed analogously to search (Table 2), with penalties asymmetrically larger than rewards to prevent exploitation. An exception disables FoV penalties during obstacle avoidance events.

**Figure 5:**
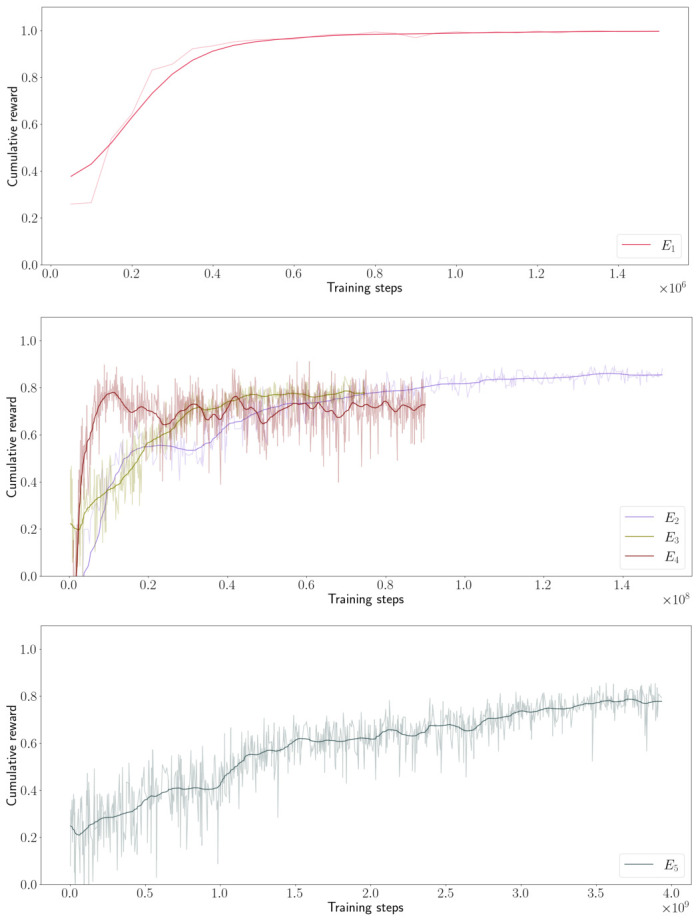
Tracking model cumulative reward training progress with heading-based control.

**Figure 6:**
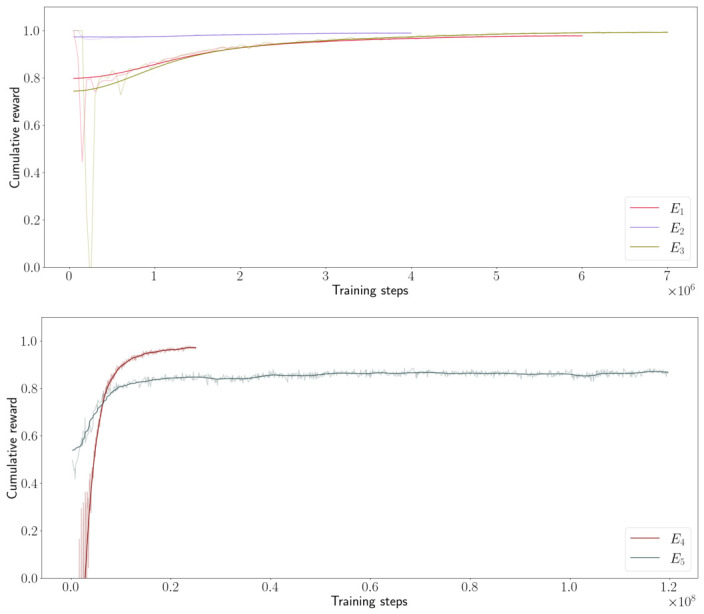
Tracking model cumulative reward training progress with waypoints-based control.

Training curves (Figure 5 and Figure 6) confirm stable convergence under curriculum learning. As for the search figures, each color corresponds to one of the previous curriculum steps. For a given color, light lines depict the raw values while the bold ones showcase an smoothed line to better identify trends.

## 6. Experiments and Results

### 6.1. Evaluation Scenario

This section describes the baseline scenario used to evaluate the proposed system architecture and the trained RL models. In contrast to the simplified training environments (designed to facilitate the acquisition of specific behaviors), the evaluation setup integrates all operational elements into a single unified scenario. It simultaneously includes search areas, mobile ground targets, and static obstacles, thereby generating a complex and dynamic environment.

All components and assumptions are consistent with those defined during training, ensuring continuity and enabling a fair assessment of generalization performance:

- The operational area, UAV initial positioning, flight dynamics, onboard sensors, navigation system, and modeling assumptions are identical to those described in Section 5.1.
- Ground vehicles act as targets for search and tracking tasks. They move continuously along straight-line trajectories connecting randomly selected waypoints within the operational boundaries. Their motion is independent of the UAV swarm, introducing dynamic and unpredictable conditions that challenge perception and tracking capabilities.
- The scenario assumes benign weather conditions, with no adverse environmental effects (e.g., rain or fog), ensuring reliable detection within sensor range.

### 6.2. Evaluation Metrics

The evaluation of the system’s performance is conducted using several metrics. It is crucial to emphasize that these metrics reflect the behavior of the swarm as a collective entity, rather than the individual actions of the drones that comprise it. The following performance metrics are employed:

- Mean revisit period: In the realm of continuous surveillance, the mean revisit period constitutes a fundamental metric that quantifies the frequency at which drones return to specific areas to update target information or identify changes. This metric is determined by partitioning the swarm’s operational area into discrete grid cells and subsequently calculating the average time each cell remains unsurveyed by any drone in the swarm, irrespective of whether the drones are engaged in search or tracking tasks. The primary objective of a swarm executing a search operation is to maximize the frequency of visits to each cell, thereby minimizing the mean revisit period and ensuring timely updates across the entire operational area.
- Target acquisition time: This metric measures the time elapsed from a target’s entry into the surveillance area to the time at which the drone swarm successfully detects and initiates tracking of the target. Efficient execution of search tasks is critical to minimizing this duration, as shorter detection times directly reflect the swarm’s ability to rapidly identify and respond to new targets within the operational area.
- Target detection rate: This metric serves as a critical indicator of the effectiveness of the drone swarm in detecting and monitoring targets within the designated area. It is quantified at each time instant by calculating the proportion of targets that remain within the field of view of the drone swarm. This value provides a dynamic measure of the swarm’s performance in maintaining situational awareness and ensuring comprehensive target coverage.
- Tracking continuity: This metric evaluates the effectiveness of the drone swarm in consistently maintaining targets within its field of view and accurately tracking their positions and movements. It is calculated as the proportion of time a target remains visible to the swarm, relative to the total duration allocated for tracking that target. The total tracking duration is defined as the interval from the target’s initial detection and assignment to a drone for monitoring, up to either the conclusion of the operation or the target’s departure from the operational area. This duration represents the timeframe during which the swarm is expected to continuously monitor the target, providing a comprehensive measure of tracking performance.
- Minimum distance to known obstacles (5th percentile): Evaluates safety margins by sampling the distance to obstacles within the proximity sensor coverage over time. For every interaction with an obstacle, the minimum separation distance recorded during that interaction is computed, where an interaction is defined as the time interval between the moment an obstacle enters the proximity sensor range and the moment it exits it. At the end of the simulation, the 5th percentile of all minimum distances (one per interaction) is computed to quantify the safety margin maintained with respect to nearby obstacles.

### 6.3. Experimental Results

This section presents the comparative evaluation of the three control methods under identical experimental conditions. For each configuration, 100 independent Monte Carlo simulations were conducted, reproducing the same conditions for each of the three methods. All scenarios were executed over a fixed horizon of 500 s to ensure strict comparability. The performance metrics, as defined in Section 6.2, assess the swarm as a collective system rather than individual drone behavior.

#### 6.3.1. Baseline Scenario

**Figure 7:**
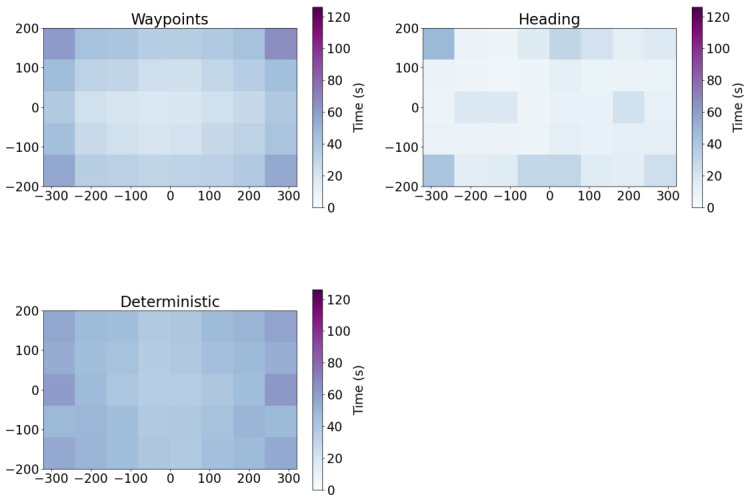
Revisit-period heatmap per method (baseline).

Search efficiency is first analyzed through the mean revisit period. Figure 7 shows the corresponding heatmaps. The heading-based strategy consistently achieves the lowest revisit times, indicating a higher temporal resolution in coverage. However, spatial heterogeneity appears near boundaries and corners, mainly due to tracking trajectories concentrating in central regions. The waypoint-based method exhibits intermediate performance, while the deterministic strategy produces the largest revisit periods and the highest variability across scenarios.

**Figure 8:**
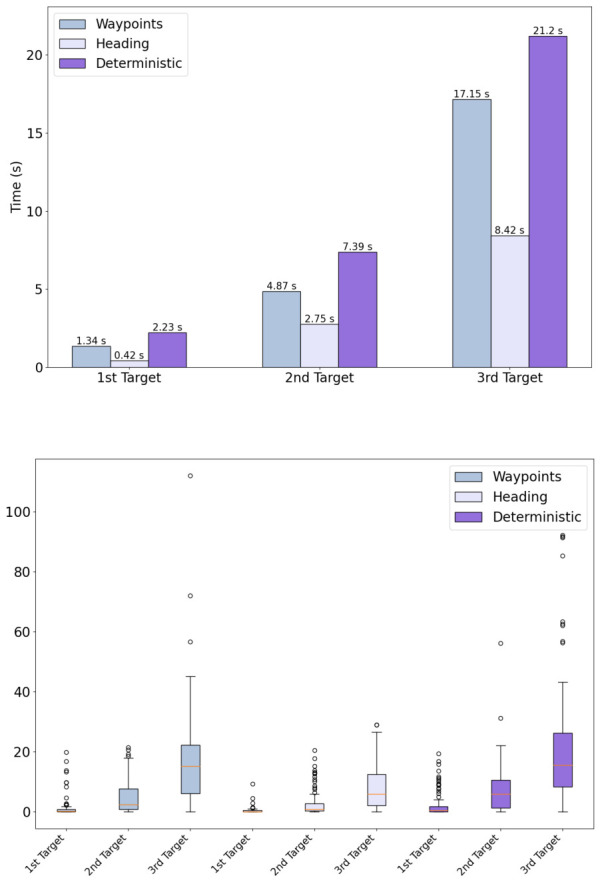
Target acquisition time per method (baseline).

Target acquisition time results are shown in Figure 8. The heading-based method consistently achieves the shortest acquisition times across all three targets, followed by the waypoint-based approach, while the deterministic strategy exhibits the longest delays. This ranking aligns with the revisit-period analysis: shorter revisit intervals directly translate into faster detection.

The box plots confirm not only lower medians for the heading-based strategy but also reduced dispersion, indicating robust performance across heterogeneous realizations.

Statistical validation was performed using a Kruskal–Wallis test. For both mean revisit time and target acquisition time, the null hypothesis of equal medians was rejected (p<0.05). Dunn–Bonferroni post hoc analysis revealed statistically significant differences for all pairwise comparisons in revisit time. For acquisition time, the heading-based method significantly outperformed the deterministic strategy for all targets, while differences between heading- and waypoint-based planning were significant for the second and third targets. Table 3, Table 4 and Table 5 detail the results.

Tracking effectiveness is evaluated through tracking continuity and detection rate. Table 6 shows that the heading-based method achieves the highest continuity, substantially outperforming the waypoint-based and deterministic strategies.

**Figure 9:**
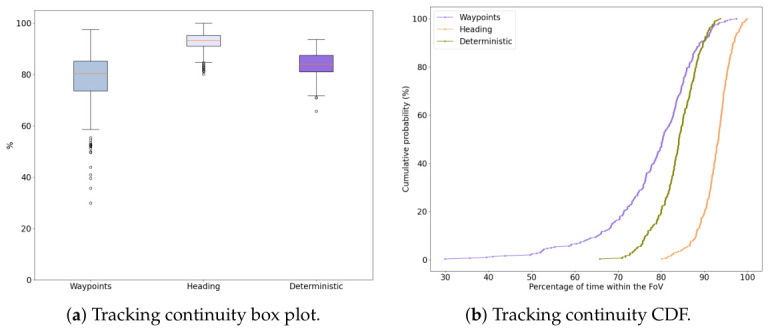
Tracking continuity per method (baseline).

The distributional analysis in Figure 9 confirms both higher medians and lower variability for the heading-based strategy. Its fine-grained control enables high-frequency directional adjustments, leading to smoother tracking and fewer interruptions. In contrast, waypoint-based and deterministic methods are constrained by predefined spatial references, limiting responsiveness to abrupt target maneuvers.

**Figure 10:**
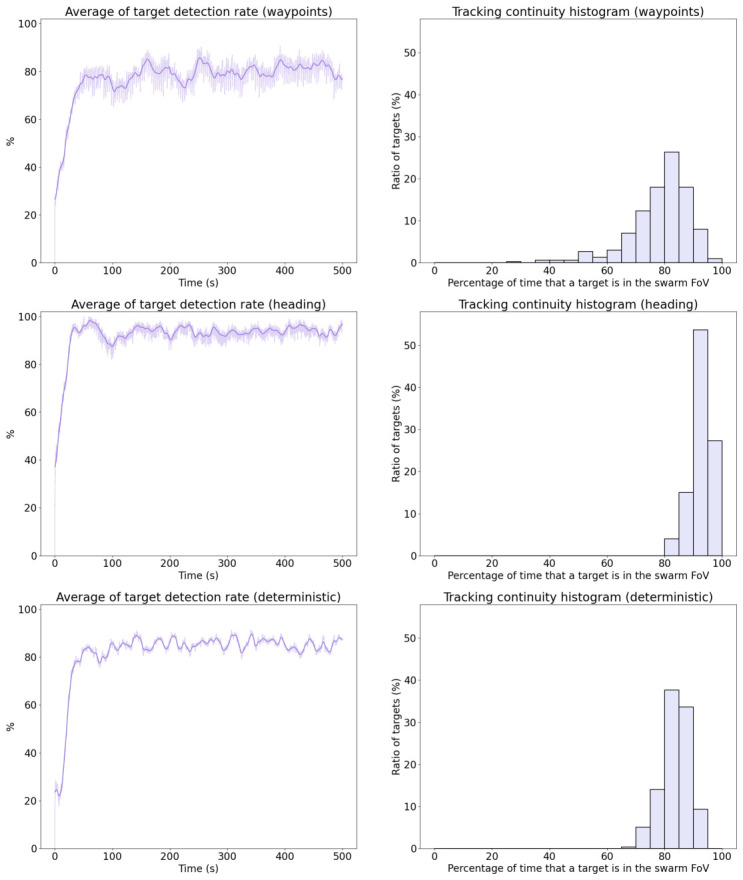
Tracking metrics per method (baseline).

Figure 10 further illustrates detection rate evolution. The heading-based method maintains a consistently higher proportion of targets within the field of view throughout the simulation horizon.

A Kruskal–Wallis test confirmed statistically significant differences in tracking continuity (p<0.05). Post hoc Dunn–Bonferroni analysis (Table 7) showed significant differences for all pairwise comparisons, confirming the superior tracking robustness of heading-based control.

#### 6.3.2. Static Obstacles Scenario

The second set of experiments introduces static obstacles while preserving all other conditions.

**Figure 11:**
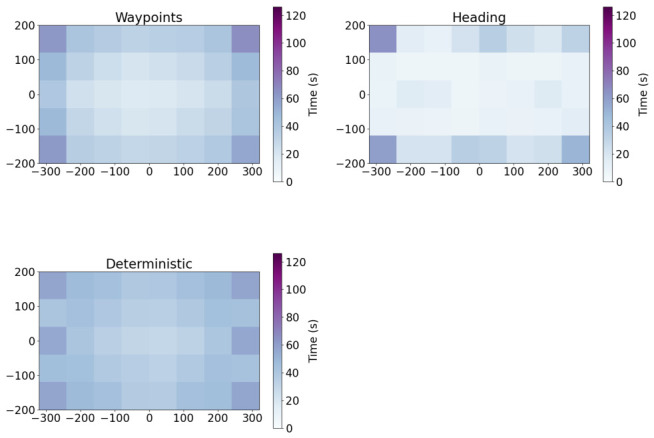
Average mean revisit period per method (static obstacles).

Revisit-period heatmaps (Figure 11) preserve the same structural patterns observed in the baseline case. Although revisit times increase slightly due to environmental constraints, the relative ranking remains unchanged, with heading-based planning achieving the most uniform coverage.

**Figure 12:**
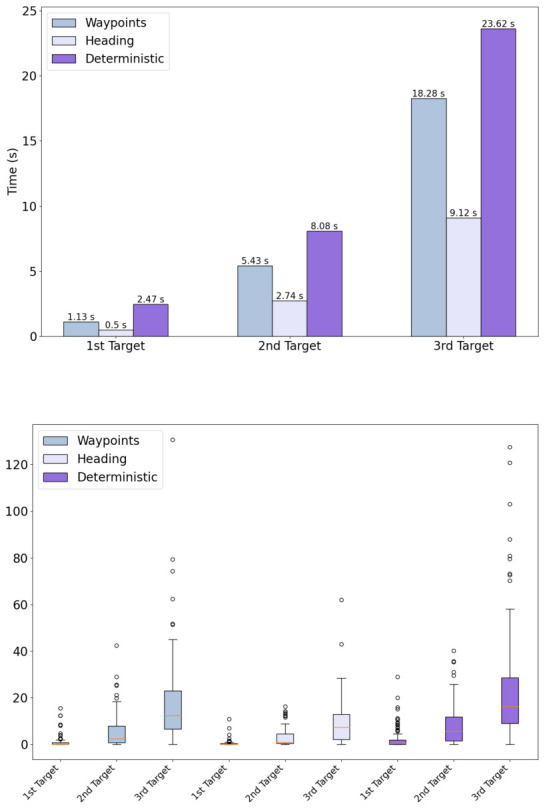
Average target acquisition time per method (static obstacles).

Target acquisition times (Figure 12) show a mild degradation for all methods, particularly for the third target. Nevertheless, the heading-based strategy maintains the shortest acquisition times. Statistical tests in Table 8 again reject the null hypothesis of equal medians, with heading-based planning significantly outperforming deterministic planning for all targets. Differences between heading- and waypoint-based methods remain significant for the second (Table 9) and third targets (Table 10).

Tracking continuity decreases for all methods due to obstacle-induced occlusions (Table 11), yet the heading-based method preserves the highest continuity (87.04%). Waypoint-based and deterministic approaches exhibit stronger degradation.

**Figure 13:**
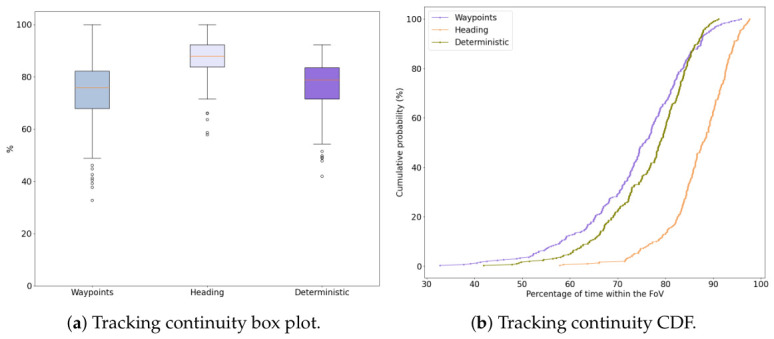
Average of tracking continuity per method (static obstacles).

**Figure 14:**
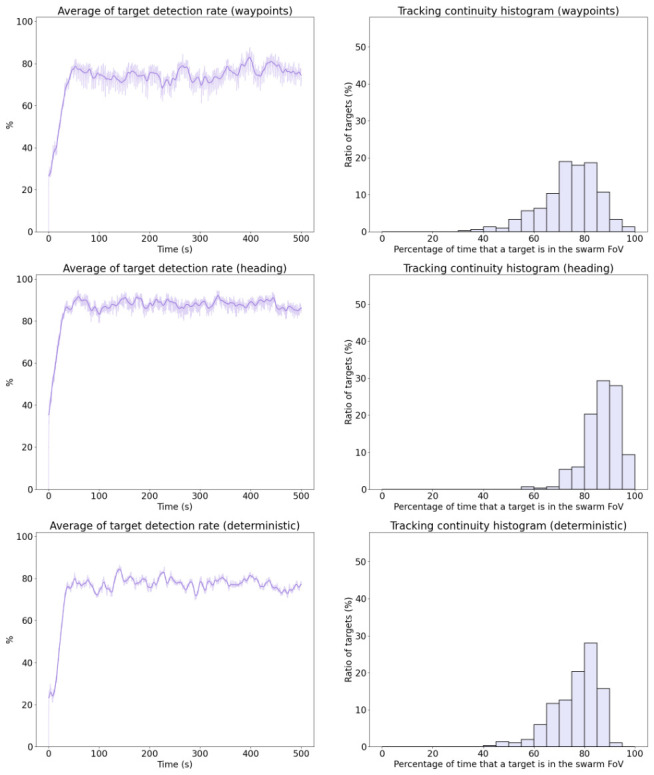
Tracking metrics per method.

Figure 13 and Figure 14 show increased variability and a downward shift in continuity distributions. However, heading-based control retains a larger proportion of high-continuity episodes.

Statistical analysis confirmed significant differences among medians (p<0.05). Post hoc results (Table 12) indicate that heading-based control significantly outperforms both alternatives, while deterministic and waypoint-based strategies become statistically comparable.

Safety performance was evaluated using the 5th percentile of the minimum distance to obstacles (Table 13). Higher values indicate larger safety margins. The heading-based method consistently maintains the greatest separation from obstacles across both search and tracking phases.

Conflict counts (Table 14) further highlight the robustness of RL-based methods, particularly heading-based control, which achieves zero conflicts during tracking.

**Figure 15:**
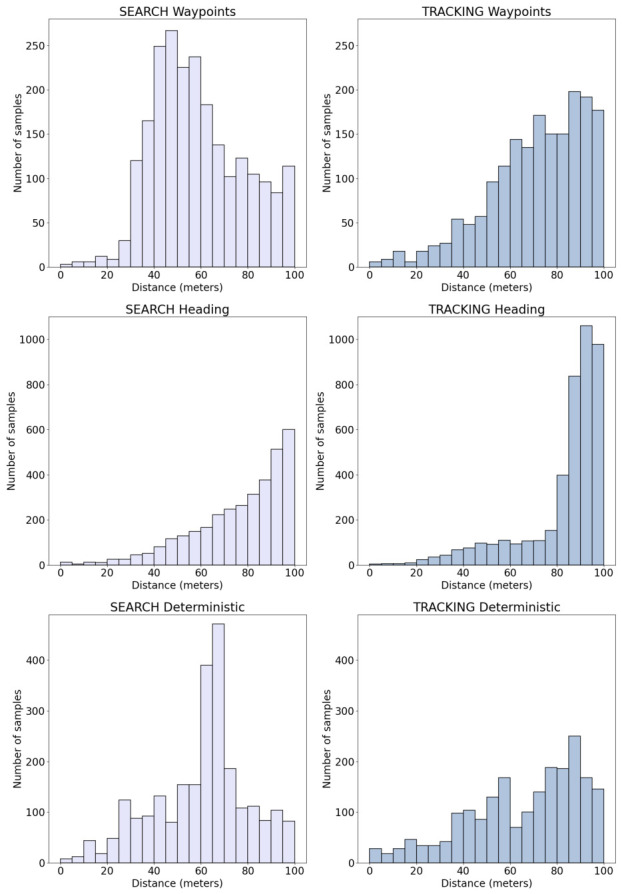
Histogram of the distance to obstacles per method.

The histogram in Figure 15 illustrates the distribution of obstacle distances. Heading-based planning shows a more uniform and consistently larger separation, whereas the deterministic strategy exhibits greater variability and closer approaches.

#### 6.3.3. Overall Discussion

Across both scenarios, the heading-based strategy consistently delivers superior search efficiency, faster target acquisition, higher tracking continuity, and stronger obstacle avoidance. The waypoint-based method achieves intermediate performance, while the deterministic approach struggles, particularly in search efficiency and safety during tracking. The results demonstrate that fine-grained, adaptive control is a decisive factor for robust swarm surveillance in both open and cluttered environments.

This superior performance stems from the combination of the heading-based action representation and its associated high-frequency control updates, both of which are intrinsic characteristics of this control paradigm. Unlike waypoint-based policies that commit to discrete targets and replan only upon arrival, heading-based agents update their velocity and direction every 0.1 s. In tracking, this allows the drone to constantly adjust its yaw and forward speed so that the target remains near the centre of the sensor’s field of view, even during abrupt target turns or occlusions. In search, the fine-grained control enables a tight, short-span zig-zag pattern: the yaw oscillates while advancing, allowing its forward-facing camera to sweep the area methodically without large displacements or wasteful detours. In addition, drones can smoothly curve around obstacles without pausing or deviating far from the coverage path. The result is a fluid, reactive behavior that maintains high revisit frequency and keeps the target visible for longer uninterrupted periods.

## 7. Conclusions and Future Work

This paper presents a systematic investigation of how different control methods shape the performance of RL-based multi-drone systems. The main contributions of this work are twofold. First (and most centrally), we provide an analysis of action-space abstractions at the control level, demonstrating that the interpretation of the policy’s output leads to fundamentally different closed-loop behaviors, with heading-based control enabling fluid, reactive trajectories that outperform discrete waypoint navigation. Second, we contribute a statistically rigorous Monte Carlo evaluation framework, supported by non-parametric hypothesis testing (Kruskal–Wallis and Dunn–Bonferroni), which conclusively shows that heading-based control achieves superior search efficiency, faster target acquisition, higher tracking continuity, and stronger obstacle avoidance in both open and cluttered environments.

In addition to these two core contributions, the paper also offers two complementary results. On the one hand, we present a unified hybrid-AI control architecture in which a single RL policy is coupled with three distinct control methods (waypoint-based, heading-based, and deterministic), enabling a fair and isolated comparison without confounding factors from different policy learning processes. On the other hand, based on extensive empirical experimentation, we derive practical design guidelines for curriculum learning and reward shaping in sparse-reward, multi-agent aerial environments, including strategies to detect and mitigate reward hacking, adapt progression to the control type, and validate each curriculum stage out-of-distribution.

Although the present study is entirely simulation-based, the proposed architecture was designed with deployment considerations in mind. A related previous work by the authors demonstrated the integration of the underlying swarm management and mission execution by physical platforms, providing an initial validation of the software architecture in a real-world deployment [40,41]. In any case, extending the evaluation to physical UAV platforms subject to aerodynamic disturbances, sensor noise, and navigation uncertainty remains an important direction for future research.

In the present study, all experiments were conducted using a fixed swarm size. The distributed control nature of the evaluated architecture implies that policy inference complexity remains constant at the individual UAV level. Future work should investigate how the observed differences between control abstractions evolve as the number of agents increases, especially for tightly coupled architectures where the coordination problem becomes more demanding. A further limitation of this study is that all RL-based methods were trained using a single set of PPO hyperparameters and curriculum-advancement criteria. While this choice was made to ensure a fair comparison focused exclusively on the control abstraction, future work should investigate the sensitivity of the observed performance differences to alternative training configurations and curriculum schedules.

Together, these contributions provide both conceptual insights into action-space design for RL-based drone control and actionable guidelines for practitioners developing modular swarm systems.

## Figures and Tables

**Table 1:** Training rewards designed for the search model.

Trigger	Reward	Explanation
Sensor center visits at least once each search area cell.	+0.9	Aimed to reach the search area, stay inside, and carry out a complete recognition of it.
Entering the search area.	+0.1	
Leaving the search area.	−0.25	
Reach max step.	−0.5	Aimed to avoid hovering/loitering positions and suboptimal behaviors.
Re-entering the operational area.	+0.1	Aimed to remain within the operational area.
Leaving the operational area.	−0.25	
Colliding with a static obstacle.	−0.5	Aimed to guarantee safety.

**Table 2:** Training rewards designed for the tracking model.

Trigger	Reward	Explanation
Reach the consecutive steps keeping the target within the agent FoV.	+0.9	Aimed to keep the target within the drone FoV.
The task target enters the sensor FoV. *	+0.1	
The task target leaves the sensor FoV. *	−0.25	
Reach max step.	−0.5	Aimed to avoid hovering/loitering positions and suboptimal behaviors.
Re-entering the operational area.	+0.1	Aimed to stay inside the operational area.
Leaving the operational area.	−0.25	
Colliding with a static obstacle.	−0.5	Aimed to guarantee safety.

* The indicated rewards are given with one exception. In cases where an obstacle is within the obstacle’s detection range at the moment the target leaves the sensor FoV, neither the target leaves sensor FoV reward nor the subsequent target enters the sensor FoV reward are given in order to ease obstacle avoidance while following the target.

**Table 3:** Dunn–Bonferroni post hoc test for the target acquisition time of the first target in baseline scenario.

	Deterministic	Heading	Waypoints
**Deterministic**	1	$3.93\times 10^{-4}$	$2.30\times 10^{-2}$
**Heading**	$3.93\times 10^{-4}$	1	0.740
**Waypoints**	$2.30\times 10^{-2}$	0.740	1

**Table 4:** Dunn–Bonferroni post hoc test for the target acquisition time of the second target in baseline scenario.

	Deterministic	Heading	Waypoints
**Deterministic**	1	$1.39\times 10^{-8}$	$3.25\times 10^{-2}$
**Heading**	$1.39\times 10^{-8}$	1	$2.77\times 10^{-3}$
**Waypoints**	$3.25\times 10^{-2}$	$2.77\times 10^{-3}$	1

**Table 5:** Dunn–Bonferroni post hoc test for the target acquisition time of the third target in baseline scenario.

	Deterministic	Heading	Waypoints
**Deterministic**	1	$1.44\times 10^{-8}$	0.747
**Heading**	$1.44\times 10^{-8}$	1	$7.77\times 10^{-6}$
**Waypoints**	0.747	$7.77\times 10^{-6}$	1

**Table 6:** Tracking continuity per method (baseline scenario).

	Waypoints	Heading	Deterministic
Tracking continuity	78.32%	92.86%	83.79%

**Table 7:** Dunn–Bonferroni post hoc test for tracking continuity in baseline scenario.

	Deterministic	Heading	Waypoints
**Deterministic**	1	$4.33\times 10^{-59}$	$5.28\times 10^{-6}$
**Heading**	$4.33\times 10^{-59}$	1	$6.11\times 10^{-98}$
**Waypoints**	$5.28\times 10^{-6}$	$6.11\times 10^{-98}$	1

**Table 8:** Dunn–Bonferroni post hoc test for the target acquisition time of the first target in static obstacle scenario.

	Deterministic	Heading	Waypoints
**Deterministic**	1	$1.95\times 10^{-4}$	$1.59\times 10^{-2}$
**Heading**	$1.95\times 10^{-4}$	1	0.681
**Waypoints**	$1.59\times 10^{-2}$	0.681	1

**Table 9:** Dunn–Bonferroni post hoc test for the target acquisition time of the second target in static obstacle scenario.

	Deterministic	Heading	Waypoints
**Deterministic**	1	$1.23\times 10^{-7}$	$4.89\times 10^{-2}$
**Heading**	$1.23\times 10^{-7}$	1	$6.14\times 10^{-3}$
**Waypoints**	$4.89\times 10^{-2}$	$6.14\times 10^{-3}$	1

**Table 10:** Dunn–Bonferroni post hoc test for the target acquisition time of the third targetin static obstacle scenario.

	Deterministic	Heading	Waypoints
**Deterministic**	1	$8.92\times 10^{-9}$	0.341
**Heading**	$8.92\times 10^{-9}$	1	$4.06\times 10^{-5}$
**Waypoints**	0.341	$4.06\times 10^{-5}$	1

**Table 11:** Tracking continuity per method (static obstacles).

	Waypoints	Heading	Deterministic
Tracking continuity	74.25%	87.04%	76.75%

**Table 12:** Dunn–Bonferroni post hoc test for tracking continuity in static obstacle scenario.

	Deterministic	Heading	Waypoints
**Deterministic**	1	$9.66\times 10^{-41}$	0.107
**Heading**	$9.66\times 10^{-41}$	1	$4.99\times 10^{-54}$
**Waypoints**	0.107	$4.99\times 10^{-54}$	1

**Table 13:** Fifth percentile of the minimum distance to obstacles.

	Waypoints	Heading	Deterministic
Search model	32.9 m	38.2 m	25.3 m
Tracking model	34.2 m	42.0 m	20.4 m

**Table 14:** Obstacle conflicts.

	Waypoints	Heading	Deterministic
Search model	2	5	4
Tracking model	3	0	10

## Data Availability

Restrictions apply to the datasets. The study relies entirely on free software (Unity ML-Agents), publicly accessible. The datasets presented in this article are not readily available because they consist of large-scale intermediate reinforcement learning logs and simulation traces that lack standalone interpretability without the exact environment configuration, random seeds, and execution context. Requests to access the datasets should be directed to the corresponding author.
